# Myocardial revascularization at extremes – surgical revascularization in acute coronary syndrome

**DOI:** 10.1186/1749-8090-8-S1-O186

**Published:** 2013-09-11

**Authors:** S Kacar, M Kacar

**Affiliations:** 1 Department for Cardiac Surgery, Institute for Cardiovascular Diseases of Vojvodina, Sremska Kamenica, Novi Sad, Serbia; 2 Department of Anesthesiology, Institute for Cardiovascular Diseases of Vojvodina, Sremska Kamenica, Novi Sad, Serbia

## Background

Patients with ACS who needs CABG are surgical challenge due to their instability, potential unrecognized co-morbidity, and the need for urgent treatment. Dual antiplatelet therapy (clopidogrel + aspirin) has to be given to almost all patients before coronarography. The same therapy becomes a threat to cardiac surgeons as it may have deleterious effects on surgical haemostasis.

## Methods

At our Institute, 122 patients with ACS were surgically revascularized using extracorporeal circulation in the first 10 days after the coronarography by one surgical team in the 5 years period. Patients were stratified into two groups: 65 patients operated within 4 days (group 1), and 57 patients operated from 5-Th to 10-Th day after the clopidogrel discontinuation (group 2). Patients who underwent reoperation, combined procedures, or off-pump revascularization were excluded.

## Results

There was no mortality in any group. Mean chest tube losses after the surgical revascularisation were 647.27 ml in group 1, and 683.67 ml in group 2. The mean quantity of RBC transfused were 639.36 ml and 470.92 ml retrospectively. The mean amount of FFP given to the group 1 was 270 ml, and to group 2 was 197.65 ml. Platelets have been given to 12 pts in the group 1, and to 1 pt in the group 2. Crioprecipitate was given to 10 pts and to 3 pts retrospectively. The median length of stay were 12.8 days in the group 1, and 18.96 days in the group 2. Detailed patient characteristics, surgical treatment, complications, and statistical analysis will be presented.

## Conclusions

The urgent and emergent surgical revascularisation using extracorporeal circulation in patients with acute coronary syndrome is safe and effective procedure. We recommend not waiting 5 or more days after the clopidogrel discontinuation. Decision of the timing of CABG has to be made after careful individual assessment of the patient clinical status, co-morbidity and angiography.

